# Inhibition of microRNA-328 Increases Ocular Mucin Expression and Conjunctival Goblet Cells

**DOI:** 10.3390/biomedicines11020287

**Published:** 2023-01-19

**Authors:** Jackson Choo, Chun-Huei Liao, Ching-Li Tseng, Jiunn-Liang Chen, Huey-Chuan Cheng, Chung-Ling Liang, Suh-Hang Hank Juo

**Affiliations:** 1 Graduate Institute of Biomedical Sciences, China Medical University, Taichung 404, Taiwan; 2 Department of Medical Research, China Medical University Hospital, Taichung 404, Taiwan; 3 Graduate Institute of Biomedical Materials and Tissue Engineering, College of Biomedical Engineering, Taipei Medical University, Taipei 110, Taiwan; 4 International Ph.D. Program in Biomedical Engineering, College of Biomedical Engineering, Taipei Medical University, Taipei 110, Taiwan; 5 Department of Ophthalmology, Kaohsiung Veterans General Hospital, Kaohsiung 813, Taiwan; 6 Department of Ophthalmology, Mackay Memorial Hospital, Taipei 104, Taiwan; 7 Bright-Eyes Clinic, Kaohsiung 800, Taiwan; 8 Dreamhawk Vision Biotech, Inc., Kaohsiung 800, Taiwan; 9 Institute of New Drug Development, China Medical University, Taichung 404, Taiwan; 10 Drug Development Center, China Medical University, Taichung 404, Taiwan

**Keywords:** anti-miR-328 oligonucleotide, dry eye disease, mucin 5AC

## Abstract

We previously reported anti-miR-328 therapy for dry eye disease (DED). Since decreased mucin secretion is a risk factor for DED, we aimed to explore whether anti-miR-328 affects mucin expression and goblet cells. MiR-328 was increased in goblet cells when they were under desiccating stress or treated with benzalkonium chloride (BAC), both of which are risk factors for DED. Based on bioinformatics tool results, miR-328 was predicted to directly target the transcription factor CREB1 that has been known to promote the expression of mucin5AC. The inhibitory effect of miR-328 on CREB1 was confirmed by the transfection assay. A miR-328 binding site on the CREB1 gene was confirmed by the luciferase assay. Furthermore, anti-miR-328 increased CREB1 and mucin5AC in cultured goblet cells according to qPCR, Western blot, and IF staining experiments. Anti-miR-328 increased mucin5AC secretion from the cultured goblet cells based on an ELISA assay for the cultured medium. Finally, impression cytology data revealed anti-miR-328 increased conjunctival goblet cells in the DED rabbits induced by BAC. In conclusion, anti-miR-328 increases CREB1 expression leading to an increase in mucin5AC production and secretion. Furthermore, anti-miR-328 also increases conjunctival goblet cells. These results warrant the further development of anti-miR-328 therapy for DED.

## 1. Introduction

Dry eye disease (DED) is a common multifactorial ocular surface disease that may cause eye discomfort, irritation, stinging, pain, and blurred vision [1]. One of the key elements contributing to the pathogenesis of DED is tear film instability, which may arise from the decreasing amount of mucin in the tear film [2,3]. According to the Tear Film and Ocular Surface Society (TFOS) Dry Eye Workshop (DEW) II Epidemiology Report, the prevalence of DED in the Asian population is 30.1%, which is higher than the 11% in Caucasians, and women have a higher risk than men [4,5,6]. For treatment, artificial tear substitutes are frequently used in patients with mild symptoms, and prescription medications such as cyclosporine (Restasis) and lifitegrast (Xiidra) are used in more severe conditions to suppress ocular inflammation [7,8]. However, the main effect of the above prescribed medications does not focus on improving tear film stability; therefore, there is a need to develop new medication to increase tear stability [9,10].

Mucin contributes to the innermost layer of tear film and plays a key role in maintaining tear film stability and protecting ocular surface integrity [11,12]. Mucins are high-molecular-weight extracellular glycoproteins that are expressed by a kind of highly specialized epithelial cell, which is the conjunctival goblet cell [13]. Among the mucin family, gel-forming mucin5AC (MUC5AC) is the most important secreted mucin on the ocular surface [14,15]. A loss of goblet cells leading to a low level of MUC5AC is a hallmark of DED [16]. We recently reported that the over-expression of microRNA-328 (miR-328) in the ocular surface is a risk factor for DED, and anti-miR-328 eye drops significantly reduce corneal staining and improve corneal re-epithelialization [17]. Since decreased mucin secretion is an important risk factor for DED [16], in the present study, we aimed to study how miR-328 affects mucin secretion.

MicroRNAs are 21–23 long non-coding single-stranded RNAs. Animal miRNA is usually bound to the 3′ untranslated region (UTR) of target genes’ mRNA. The annealing of miRNA to the 3′UTR will cause the inhibition of protein translation and/or the cleavage of target genes’ mRNA. miRNA regulates cell growth, differentiation, and apoptosis; thus, the dysregulation of miRNAs may lead to diseases, including DED [18,19].

First, we conducted this study to explore how miR-328 affects mucin secretion. In this study, we demonstrated that cAMP response element-binding protein1 (CREB1) is a miR-328 target. CREB1 is a transcription factor and has been reported to bind and mediate MUC5AC gene expression [20,21,22]. We then examined the effect of anti-miR-328 on MUC5AC secretion and ocular goblet cells using in vitro and in vivo models, respectively.

## 2. Materials and Methods

### 2.1. Cell Culture

The Statens Seruminstitut Rabbit Cornea (SIRC) cell line was purchased from the Bioresource Collection and Research Center (BCRC). The cells were cultured in Dulbecco’s modified Eagle medium (DMEM) (5 mM d-(+)-glucose; Gibco, Thermo Fisher Scientific, Inc., Waltham, MA, USA), supplemented with 10% fetal bovine serum and 100 U/mL of penicillin at 37 °C in an atmosphere of 5% CO_2_ and passaged every 3–5 days.

Rabbit conjunctival goblets cells were isolated as previously described with slight modifications [23,24]. Briefly, rabbits were euthanized, and the eyeballs were removed and placed in ice-cold PBS. The entire conjunctiva was trimmed, freed of extraneous tissues, and placed in a sterile petri dish containing ice-cold PBS and 300 µg/mL penicillin–streptomycin. Tissues were washed using ice-cold PBS containing 300 µg/mL penicillin–streptomycin, minced into 1-mm^3^ pieces, and anchored onto six-well culture dishes. The explants were cultured in RPMI-1640 (1 mM sodium pyruvate, 10 mM HEPES, Gibco, Thermo Fisher Scientific, Inc., Waltham, MA, USA), supplemented with 10% fetal bovine serum, 1X nonessential amino-acid mixture (Gibco, Thermo Fisher Scientific, Inc., Waltham, MA, USA), and 100 U/mL of penicillin at 37 °C in an atmosphere of 5% CO_2_. Cells were permitted to grow from the tissue explant for 14 days until reaching 85% confluence, and then the explants were removed and discarded. The successfully isolated conjunctival goblet cells were passaged every 3–5 days.

### 2.2. Induction of Desiccation Stress

Goblet cells with a density of 1 × 10^5^ cells/well were seeded on the membrane of 6-well hanging transwell inserts with 0.4 µm pore size (Merck Millipore, Burlington, MA, USA) and cultivated for 24 h to allow the cells to attach to the membrane. For the induction of desiccation stress, the medium in the upper layer of the membrane was aspirated and cells were washed with phosphate-buffered saline (PBS). The medium in the lower layer of the membrane was changed to fresh medium containing anti-miR-328 or vehicle, and the cells were incubated for another 24 h.

### 2.3. Anti-miR-328 Eye Drops

The description of eye drops containing anti-miR-328 oligonucleotide can be found elsewhere [17]. In brief, the composition of the anti-miR-328 eye drops is a single-stranded anti-miR-328 oligonucleotide that perfectly matches to the seed region of miR-328. This oligonucleotide was dissolved in phosphate-buffered saline (PBS) to serve as eye drops in the present study.

### 2.4. miRNA Targets and Pathway Prediction

We used TargetScan [25] (v7.2, https://www.targetscan.org/vert_72/, accessed on 10 March 2022) to investigate the target genes of miR-328. Ingenuity Pathway Analysis (IPA, QIAGEN, Germantown, MD, USA) was used to predict the downstream molecular pathway of miR-328 which is related to DED.

### 2.5. MicroRNA Transfection Assay

SIRC cells were seeded on a 12-well culture plate with a density of 1.2 × 10^5^ cells/well. After 24 h, different concentrations of miR-328 mimic (Phalanx Biotech, Zhubei City, Hsinchu Country, Taiwan) were transfected into cells using Hi-Perfect reagent (QIAGEN, Germantown, MD, USA) according to the manufacturer’s protocol. After 24 h, cells were harvested for RNA extraction.

### 2.6. Construction of Reporter Plasmids

We first synthesized double-stranded oligonucleotides that contain 20 bp surrounding the miR-328 target site on CREB1 3′UTR in three tandem copies and used the restriction enzymes Nhel and Xhol to construct the cloning sites. The oligonucleotides were cloned into a reporter vector (pmirGLO Dual-Luciferase miRNA Target Expression Vector, Promega, Madison, WI, USA) by T4 DNA ligase (New England BioLabs, Ipswich, MA, USA). One reporter construct carried a wild-type sequence and the other carried a mutant sequence. All constructs were confirmed by DNA sequencing (see primers in Supplementary Table S1).

### 2.7. Transient Transfection and Luciferase Reporter Assay

Human embryonic kidney 293 cells (HEK-293) were purchased from ATCC and cultured in Dulbecco’s modified Eagle medium (DMEM) (5 mM d-(+)-glucose; Gibco, Thermo Fisher Scientific, Inc., Waltham, MA, USA), supplemented with 10% fetal bovine serum at 37 °C in an atmosphere of 5% CO_2_. The cells were seeded on a 96-well culture plate with a density of 1.5 × 10^4^ cells/well. After 24 h, miR-328 mimic (Phalanx Biotech, Zhubei City, Hsinchu Country, Taiwan) was transfected into cells using Hi-Perfect reagent (QIAGEN, Germantown, MD, USA), and the constructs were co-transfected into cells using LipofectamineTM 2000 Transfection Reagent (Invitrogen, Thermo Fisher Scientific, Inc., Waltham, MA, USA). After 24 h, the cells were lysed, and the luciferase activity was measured using the Dual-Luciferase Reporter^®^ Assay System (Promega, Madison, WI, USA) and Fluoroskan Ascent FL (Thermo Fisher Scientific, Waltham, MA, USA).

### 2.8. Reverse Transcription and Quantitative Polymerase Chain Reaction

RNA was extracted using the GENEzol^TM^ TriRNA Pure Kit (Geneaid, Xizhi Dist., New Taipei City, Taiwan). RNA was reversely transcribed into first-strand cDNA using the High-Capacity RNA-to-cDNA™ Kit (Applied Biosystem^TM^, Foster City, CA, USA) according to the manufacturer’s protocol. A quantitative polymerase chain reaction was performed using the Fast SYBR™ Green Master Mix (Applied Biosystem^TM^, Foster City, CA, USA). Results were normalized to the CT value of GAPDH, and the relative fold expression was calculated using the 2^–∆∆Ct^ method. The primers are shown in Supplementary Table S2.

### 2.9. Western Blotting

Whole-cell extracts were prepared using the RIPA buffer. Equal amounts of total protein were used for Western blotting. Primary antibodies used in this study were confirmed to react with rabbit protein, including MUC5AC (Clone 45M1, MA5-12178, Invitrogen, Thermo Fisher Scientific, Inc., Waltham, MA, USA) and beta-actin (ab8227, Abcam, Waltham, Boston, MA, USA). All images were captured using ChemiDocTM Imaging System (BioRad, Hercules, CA, USA). The gray intensity of protein blots was measured using ImageJ software (NIH, Bethesda, MD, USA).

### 2.10. Immunofluorescence Staining

Goblet cells were seeded on 24-well culture plates with a density of 1 × 10^5^ cells/well. After 24 h, cells were treated with 0.02% BAC (Sigma-Aldrich, St. Louis, MO, USA). After 10 min, the culture medium was replaced with fresh medium containing anti-miR-328, and cells were incubated for a further 24 h. Then, culture medium was removed, and cells were fixed in 4% paraformaldehyde blocked with 0.5% Triton X-100/5% bovine serum albumin for 2 h at room temperature. The samples were incubated with primary antibodies overnight at 4 °C and then with secondary antibodies for 1 h at room temperature. The primary antibody used in this study was mouse monoclonal antibody against MUC5AC (Clone 45M1, MA5-12178, Invitrogen, Thermo Fisher Scientific, Inc., Waltham, MA, USA). The secondary antibody used in this study was goat-anti-mouse IgG antibody (SAB4600105, Sigma-Aldrich, St. Louis, MO, USA). All images were captured using an inverted microscope (Leica DMi8, Leica, Wetzlar, Hessen, Germany).

### 2.11. ELISA

Goblet cells were seeded on 6 cm^2^ culture dishes with a density of 1.2 × 10^6^ cells/dish. After 24 h, cells were treated with 0.02% BAC (Sigma-Aldrich, St. Louis, MO, USA). After 10 min, the culture medium was replaced with fresh medium containing anti-miR-328, and cells were incubated for a further 48 h. After 48 h, the culture medium was collected, and ELISA was performed using the Rabbit MUC5AC ELISA Kit (FineTest^®^, Wuhan, Hubei, China) according to the manufacturer’s procedure.

### 2.12. Impression Cytology and Periodic Acid–Schiff (PAS) Staining

All rabbits were adopted a week before the experiments in the Laboratory Animal Center of China Medical University (Taiwan, ROC). The animals were kept at a controlled temperature (23 ± 2 °C), in relative humidity (60% ± 10%), with 12 h light–dark cycles (07:00–19:00), and given food and water ad libitum. All procedures were approved by the Institutional Animal Care and Use Committee (IACUC no. 2021-276) of China Medical University. A total of 30 rabbits were used for the impression cytology study. The rabbits were randomly divided into 3 groups: the normal control group (*n* = 10), the vehicle group (*n* = 10), and the anti-miR-328 group (*n* = 10). Ten rabbits in the normal control were not treated with anything. DED was induced in the other 20 rabbits from days 1 to 21 by instilling 20 µL of 0.15% BAC twice per day (9 am and 5 pm). A quantity of 20 µL of anti-miR-328 (160 µM) or vehicle (PBS) was instilled in both eyes twice per day from day 8 to day 21, whereas BAC was still instilled 10 min after anti-miR-328 or vehicle treatment in these 2 weeks. Conjunctival impression cytology specimens were collected on day 21. After instilling 0.5% Alcaine and wiping away excessive fluid from the eye, a half-circular piece of nitrocellulose filter paper (T ADVANTEC^®^, Chiyoda-ku, Tokyo, Japan) with a diameter of 5.5 mm was placed on the superior bulbar conjunctiva. The filter paper was held in place for 1 min via slight pressure and was then peeled off from the eye and immediately fixed with 10% neutral buffered formalin. Periodic acid–Schiff (PAS) staining was performed using the PAS Stain Kit (ScyTek, Logan, UT, USA) according to the manufacturer’s protocol. The number of goblet cells was counted under a microscope at a 400× magnification. The density of goblet cells was quantified and expressed as the average number of cells in three random fixed areas (0.36 mm^2^) of each specimen in a high-powered field.

### 2.13. Statistical Analysis

Data are presented as the mean ± standard error of the mean. Student’s *t*-test was used to compare the different groups (i.e., anti-miR-328 vs. vehicle). A *p* value of less than 0.05 was considered statistically significant in all experiments. Data analysis and figure plotting were performed using Prism 8 software (GraphPad Software Inc., San Diego, CA, USA).

## 3. Results

### 3.1. CREB1 Is a Direct Target of miR-328

**Figure 1:**
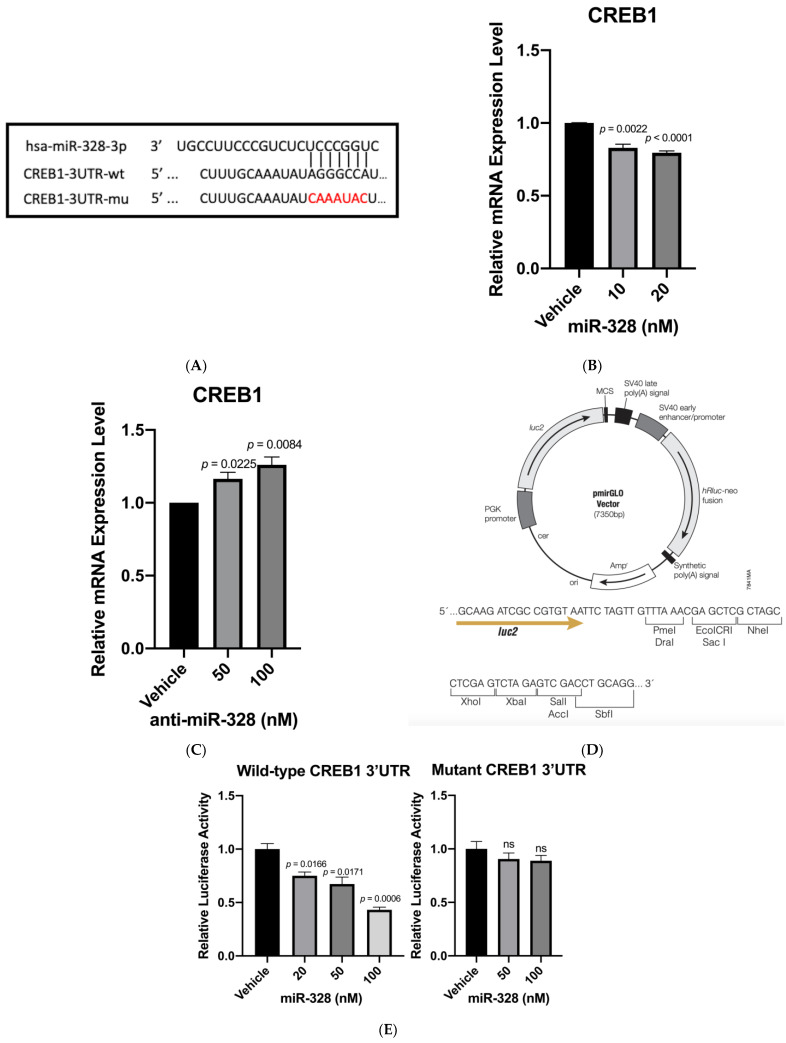
Identification of *CREB1* as a miR-328 target. (**A**) The wild-type and mutant sequences at the miR-328 binding site on *CREB1* 3′UTR. wt = wild-type; mu = mutant. (**B**) MiR-328 knocks down the *CREB1* RNA expression level. *n* = 3 for each group. (**C**) Anti-miR-328 increases *CREB1* RNA levels. *n* = 3 for each group. (**D**) The commercially available vector used in the luciferase study to carry either wild-type or mutant 3′UTR. (**E**) MiR-328 knocks down the luciferase activity in the cells containing plasmid vectors with wild-type *CREB1* 3′UTR but not mutant *CREB1* 3′UTR. *n* = 3 for each group.

We first used TargetScan (v7.2) [25] to predict any miR-328 binding sites on the MUC5AC gene. The result show that MUC5AC is not a miR-328 direct target. Then, the Ingenuity Pathway Analysis (IPA, QIAGEN, Germantown, MD, USA) software was employed to search for any relationship between miR-328 and MUC5AC, and the results indicate that miR-328 may indirectly inhibit the MUC5AC gene via CREB1. Furthermore, the data from TargetScan also suggested a miR-328 binding site on the 3′UTR of the CREB1 gene (Figure 1A). Since CREB1 was reported to directly increase MUC5AC expression [20,21], we then conducted a series of studies to confirm miR-328/CREB1/MUC5AC signaling.

### 3.2. Confirmation of CREB1 as a miR-328 Direct Target

A transfection study was conducted to demonstrate that miR-328 inhibits the CREB1 gene expression in SIRC cells. The results indicate that miR-328 mimic significantly inhibited CREB1 expression (Figure 1B). We also demonstrated that anti-miR-328 increased CREB1 expression in the goblet cells transiently treated with 0.02% BAC (Figure 1C). Such a result further confirmed that miR-328 regulated CREB1 levels. A luciferase reporter assay was conducted to provide evidence of direct binding of miR-328 to 3′UTR of CREB1. We created two different constructs using pmirGLO plasmid vector (Figure 1D): one carried three tandem copies of wild-type CREB1 3′UTR sequence, and the other carried three tandem copies of mutant sequence (Figure 1A). After the co-transfection of miR-328 mimic and plasmid constructs for 24 h, the luciferase assay showed that miR-328 dose-dependently inhibited luciferase activity in the cells containing constructs with wild-type CREB1 3′UTR. On the contrary, miR-328 barely had an effect on inhibiting luciferase activity in the cells containing constructs with mutant 3′UTR (Figure 1E).

### 3.3. Anti-miR-328 Increases MUC5AC Expression in Goblet Cells

**Figure 2:**
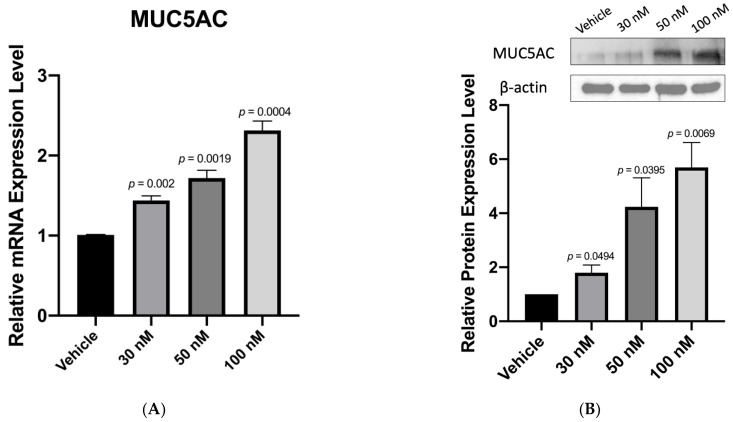

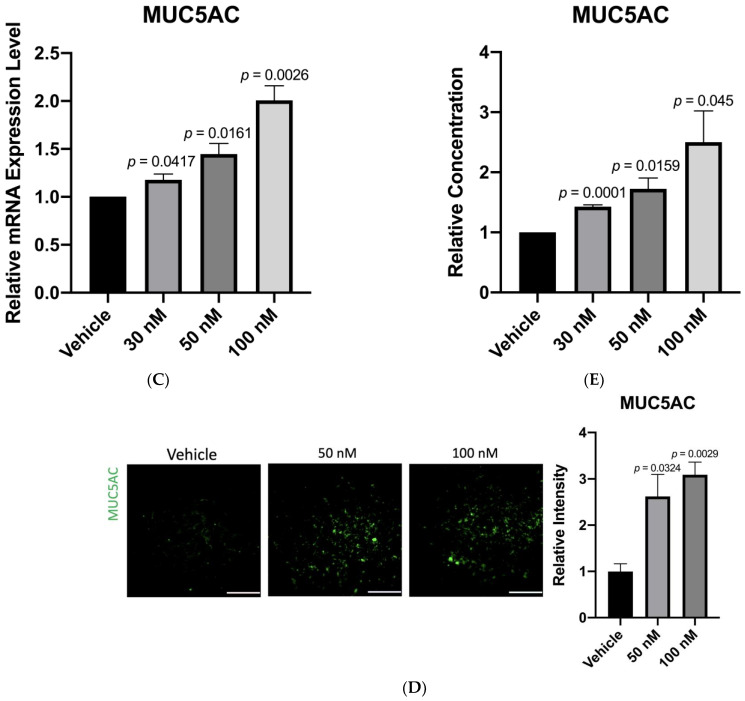
Anti-miR-328 treatment increases MUC5AC expression: (**A**,**B**) In BAC-treated rabbit goblet cells, anti-miR-328 dose-dependently increases MUC5AC RNA according to quantitative PCR and protein expression by Western blotting, *n* = 3 for each group. (**C**) Quantitative PCR results reveal anti-miR-328 dose-dependently increases MUC5AC RNA in rabbit goblet cells exposed to desiccation stress, *n* = 3 for each group. (**D**) Immunofluorescence of rabbit goblet cells reveals increased MUC5AC signals with anti-miR-328 treatment. The right panel is the quantitative data of immunofluorescence. Magnification: 100×. Scale bar = 200 µm, *n* = 3 for each group. (**E**) ELISA results reveal a dose-dependent increase in MUC5AC secretion with anti-miR-328 treatment in the culture medium of BAC-treated goblet cells. *n* = 3 for each group.

Given that anti-miR-328 increased the CREB1 expression level, and CREB1 was reported to promote MUC5AC expression in bronchial epithelial cells [20], we further tested if anti-miR-328 would increase MUC5AC expression in goblet cells. Our data show that anti-miR-328 dose-dependently increased MUC5AC RNA (Figure 2A) and that protein levels (Figure 2B) in rabbit goblet cells were transiently exposed to BAC. Similarly, anti-miR-328 treatment increased MUC5AC RNA in the goblet cells subjected to desiccation stress (Figure 2C). The positive effect of anti-miR-328 on MUC5AC expression was also demonstrated by immunofluorescence (IF) staining in BAC-treated goblet cells (Figure 2D). To test whether anti-miR-328 also promotes the secretion of MUC5AC from the goblet cells, an ELISA assay was used to measure MUC5AC protein levels in the medium of goblet cell culture. Our results show that anti-miR-328 dose-dependently increased MUC5AC secretion in BAC-treated goblet cells (Figure 2E).

### 3.4. Anti-miR-328 Treatment Increases Goblet Cells in Rabbit Eyes

**Figure 3:**
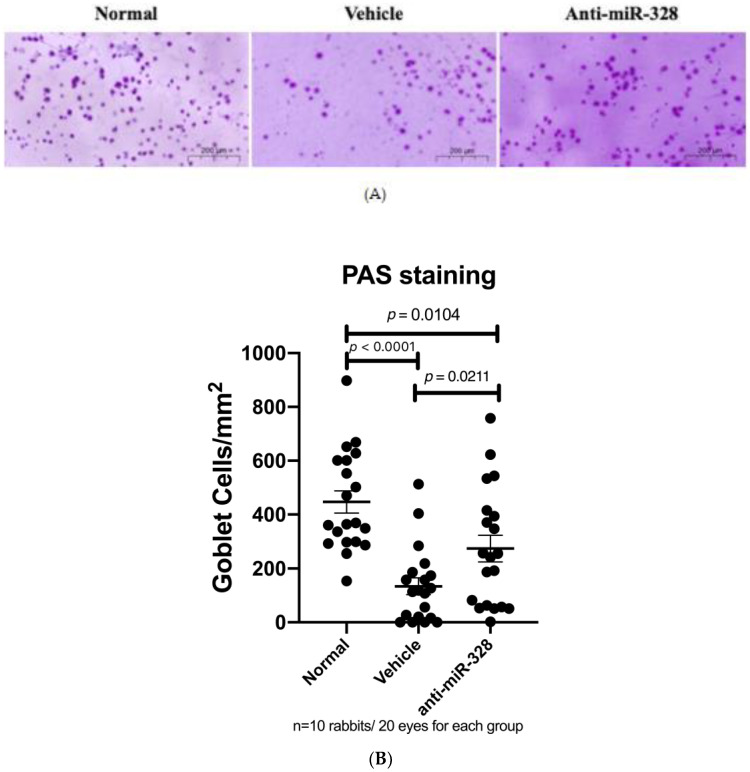
Anti-miR-328 treatment increases goblet cells. (**A**) Representative images of PAS staining, scale bar = 200 µm. (**B**) PAS staining results show that anti-miR-328 treatment increased conjunctival goblets cells in BAC-induced DED rabbits. *n* = 10 rabbits/20 eyes for each group.

An in vivo study was conducted to confirm our findings obtained from the in vitro studies. There were 30 rabbits in the following three groups: normal control, and DED rabbits with anti-miR-328 or vehicle treatment. Impression cytology on ocular surfaces and periodic acid–Schiff (PAS) staining were performed to evaluate the numbers of mucin-positive goblet cells. Our results show that the number of goblet cells was decreased in the DED rabbits. Compared with the normal group, the anti-miR-328 group had many more goblet cells than the vehicle group (mean ± SEM: 446.7 ± 41.3, 134.1 ± 31.2, and 273.8 ± 49.0 for the normal group, vehicle group, and anti-miR-328 group, respectively). Anti-miR-328 treatment significantly increased the number of goblet cells compared to vehicle treatment (Figure 3B; *p* = 0.0211). The results from the animal study validated that anti-miR-328 treatment increases MUC5AC on the ocular surface.

## 4. Discussion

**Figure 4:**
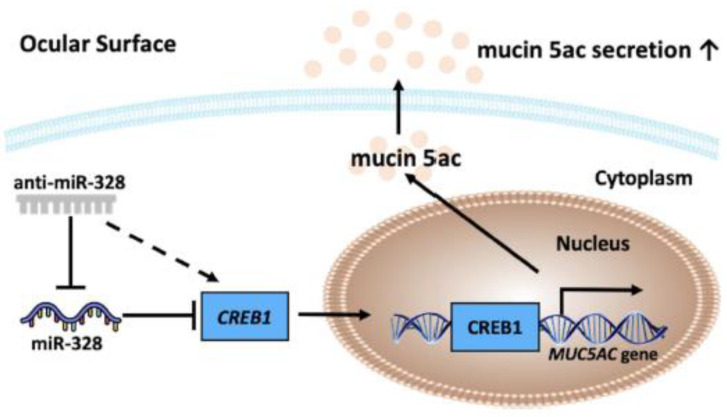
Schematic diagram shows the mechanism of anti-miR-328 for MUC5AC expression.

Our previous study demonstrated that miR-328 is a risk factor for DED development [17]. In the present study, we aimed to illustrate the role of miR-328 in terms of MUC5AC expression (Figure 4). We first demonstrated that miR-328 directly targeted and inhibited CREB1 expression levels. Consistently, anti-miR-328 oligonucleotide increased CREB1 levels in rabbit conjunctival goblet cells. CREB1 is a transcription factor that has been reported to promote MUC5AC expression [20,21]. To further confirm the effect of anti-miR-328 on MUC5AC expression, the impression cytology of conjunctiva was conducted in rabbits with DED. The results show that rabbits receiving anti-miR-328 eye drops had more goblet cells than those receiving vehicle treatment. Our results indicate that the therapeutic effects of anti-miR-328 in DED animals in our previous study [17] may be also attributed to promoting ocular MUC5AC secretion, leading to an increase in tear film stability. Figure 4 shows a schematic diagram of the mechanism of miR-328/anti-miR-328 in relation to MUC5AC expression.

A decrease in MUC5AC secretion has been reported in patients with dry eye [15]. Secreted MUC5AC contributes to tear fluid stability because of its ability to promote water retention on the ocular surface for long periods. In addition, MUC5AC might suppress epithelial damage by lubricating the ocular surface. Epidemiological studies showed that the prolonged use of computers was associated with a low MUC5AC concentration in users’ tears without a reduction in tear secretion [26]. Therefore, there is a need to develop drugs that increase mucin expression and secretion. We recently reported that anti-miR-328 therapy increased corneal healing, reduced apoptosis of corneal cells, and decreased the obstruction of Meibomian glands [17]. The present study adds another mechanism of action of anti-miR-328 to support its potential in treating DED.

The 3% diquafosol ophthalmic solution, Diquas^®^, has been approved to treat DED in some Asian countries. Diquafosol is a mucin secretagogue. Diquafosol is a P2Y2 receptor agonist [27] that initiates a signaling pathway to increase intracellular Ca^2+^ concentrations, which leads to the secretion of mucin stored in the secretory granules in a relatively short period of time [28]. However, anti-miR-328 may use a different mechanism to influence MUC5AC expression levels. By neutralizing miR-328, anti-miR-328 increases the transcription factor CREB1, which promotes the expression of the MUC5AC gene. In addition, anti-miR-328 treatment increases the number of conjunctival goblet cells. Therefore, anti-miR-328 may have a long-lasting therapeutic effect because it increases MUC5AC gene expression.

There are some strengths and weaknesses in this study. First, both in vitro and in vivo models were used to demonstrate consistent results for anti-miR-328. Only a few studies have investigated whether microRNAs regulate mucin expression. Based on our finding, it is worthwhile to study this under-researched topic, which may lead to the development of new therapeutic interventions in mucin-related diseases. Notably, mucins are secreted in multiple organs, such as the liver, lung, gut, and kidney, and they are also associated with cancer metastasis [29,30]. There are some limitations in the present study. DED is a complex disease with various etiologies. We only used BAC to induce DED in the animal study, which may not fully recapitulate the disease in humans. However, we previously have shown that both BAC and hyperosmotic condition induced miR-328 expression in corneal cells [17]. Therefore, the effect of anti-miR-328 demonstrated in the present study is unlikely to be restricted to BAC and desiccation-stress-induced dry eye. Although MUC5AC is the primary mucin on the ocular surface, the effects of miR-328 on other ocular mucins will need to be explored in the future.

## 5. Conclusions

This study is the first report to reveal that anti-microRNA therapy may possess a promising approach to altering mucin expression. We showed that anti-miR-328 increased CREB1 expression, leading to an increase in MUC5AC production and secretion. Anti-miR-328 also increased conjunctival goblet cells. All of these results warrant the further development of anti-miR-328 therapy for dry eye disease.

## 6. Patents

C.L.L. and S.H.H.J. filed a patent for anti-miR-328 therapy for dry eye disease.

## Data Availability

Not applicable.

## Supplementary Materials

The following supporting information can be downloaded at: https://www.mdpi.com/article/10.3390/biomedicines11020287/s1, Table S1: Primers used for plasmid construction. Table S2: Primers used in the real-time PCR experiments. Figure S1: Desiccation stress increased miR-328 expression in goblet cells.

## References

[1] Yazdani M., Elgstøen K.B.P., Rootwelt H., Shahdadfar A., Utheim Ø.A., Utheim T.P. (2019). Tear Metabolomics in Dry Eye Disease: A Review. Int. J. Mol. Sci..

[2] Craig J.P., Nelson J.D., Azar D.T., Belmonte C., Bron A.J., Chauhan S.K., de Paiva C.S., Gomes J.A.P., Hammitt K.M., Jones L. (2017). TFOS DEWS II Report Executive Summary. Ocul. Surf..

[3] Davidson H.J., Kuonen V.J. (2004). The tear film and ocular mucins. Vet. Ophthalmol..

[4] Stapleton F., Alves M., Bunya V.Y., Jalbert I., Lekhanont K., Malet F., Na K.S., Schaumberg D., Uchino M., Vehof J. (2017). TFOS DEWS II Epidemiology Report. Ocul. Surf..

[5] Tian Y.J., Liu Y., Zou H.D., Jiang Y.J., Liang X.Q., Sheng M.J., Li B., Xu X. (2009). Epidemiologic study of dry eye in populations equal or over 20 years old in Jiangning District of Shanghai. Zhonghua Yan Ke Za Zhi.

[6] Viso E., Rodriguez-Ares M.T., Gude F. (2009). Prevalence of and associated factors for dry eye in a Spanish adult population (the Salnes Eye Study). Ophthalmic Epidemiol..

[7] Messmer E.M. (2015). The pathophysiology, diagnosis, and treatment of dry eye disease. Dtsch. Ärzteblatt Int..

[8] Abidi A., Shukla P., Ahmad A. (2016). Lifitegrast: A novel drug for treatment of dry eye disease. J. Pharmacol. Pharmacother..

[9] Dunn J.D., Karpecki P.M., Meske M.E., Reissman D. (2021). Evolving knowledge of the unmet needs in dry eye disease. Am. J. Manag. Care.

[10] Sullivan D.A., Hammitt K.M., Schaumberg D.A., Sullivan B.D., Begley C.G., Gjorstrup P., Garrigue J.S., Nakamura M., Quentric Y., Barabino S. (2012). Report of the TFOS/ARVO Symposium on global treatments for dry eye disease: An unmet need. Ocul. Surf..

[11] Mantelli F., Argüeso P. (2008). Functions of ocular surface mucins in health and disease. Curr. Opin. Allergy Clin. Immunol..

[12] Baudouin C., Rolando M., Benitez Del Castillo J.M., Messmer E.M., Figueiredo F.C., Irkec M., Van Setten G., Labetoulle M. (2019). Reconsidering the central role of mucins in dry eye and ocular surface diseases. Prog. Retin. Eye Res..

[13] Gipson I.K., Argüeso P. (2003). Role of mucins in the function of the corneal and conjunctival epithelia. Int. Rev. Cytol.

[14] Xu K., Liu X.N., Zhang H.B., Zhu X.P., Zhang X.J. (2022). Tear film instability is associated with weakened colocalization between occludin and MUC5AC in scopolamine-induced dry eye disease (DED) rats. Int. Ophthalmol..

[15] Hori Y. (2018). Secreted Mucins on the Ocular Surface. Invest. Ophthalmol. Vis. Sci..

[16] Portal C., Gouyer V., Gottrand F., Desseyn J.L. (2019). Ocular mucins in dry eye disease. Exp. Eye Res..

[17] Liao C.H., Tseng C.L., Lin S.L., Liang C.L., Juo S.H. (2022). MicroRNA Therapy for Dry Eye Disease. J. Ocul. Pharmacol. Ther..

[18] Ambros V. (2004). The functions of animal microRNAs. Nature.

[19] Treiber T., Treiber N., Meister G. (2019). Regulation of microRNA biogenesis and its crosstalk with other cellular pathways. Nat. Rev. Mol. Cell Biol..

[20] Chung W.C., Ryu S.H., Sun H., Zeldin D.C., Koo J.S. (2009). CREB mediates prostaglandin F2alpha-induced MUC5AC overexpression. J. Immunol..

[21] Chen Y., Garvin L.M., Nickola T.J., Watson A.M., Colberg-Poley A.M., Rose M.C. (2014). IL-1β induction of MUC5AC gene expression is mediated by CREB and NF-κB and repressed by dexamethasone. Am. J. Physiol. Lung Cell Mol. Physiol..

[22] Wen A.Y., Sakamoto K.M., Miller L.S. (2010). The role of the transcription factor CREB in immune function. J. Immunol..

[23] Shatos M.A., Rios J.D., Tepavcevic V., Kano H., Hodges R., Dartt D.A. (2001). Isolation, characterization, and propagation of rat conjunctival goblet cells in vitro. Invest. Ophthalmol. Vis. Sci..

[24] Saha P., Kim K.J., Lee V.H. (1996). A primary culture model of rabbit conjunctival epithelial cells exhibiting tight barrier properties. Curr. Eye Res..

[25] Agarwal V., Bell G.W., Nam J.W., Bartel D.P. (2015). Predicting effective microRNA target sites in mammalian mRNAs. Elife.

[26] Uchino Y., Uchino M., Yokoi N., Dogru M., Kawashima M., Okada N., Inaba T., Tamaki S., Komuro A., Sonomura Y. (2014). Alteration of tear mucin 5AC in office workers using visual display terminals: The Osaka Study. JAMA Ophthalmol..

[27] Lau O.C., Samarawickrama C., Skalicky S.E. (2014). P2Y2 receptor agonists for the treatment of dry eye disease: A review. Clin. Ophthalmol..

[28] Nakamura M., Imanaka T., Sakamoto A. (2012). Diquafosol ophthalmic solution for dry eye treatment. Adv. Ther..

[29] Ali A.S., Ajaz A. (2017). The role of mucin-educated platelet activation in tumor invasiveness: An unfolding concern in the realm of cancer biology. BioMedicine.

[30] Kufe D.W. (2009). Mucins in cancer: Function, prognosis and therapy. Nat. Rev. Cancer.

