# Supplementary material for: Inhibition of microRNA-328 Increases Ocular Mucin Expression and Conjunctival Goblet Cells
Source: Biomedicines. 2023 Jan 19;11(2):287. doi: 10.3390/biomedicines11020287 (PMC9953597; doi:10.3390/biomedicines11020287)
Supplement: Supplementary file 1 [file biomedicines-11-00287-s001.zip › Supplementary figure.pdf]

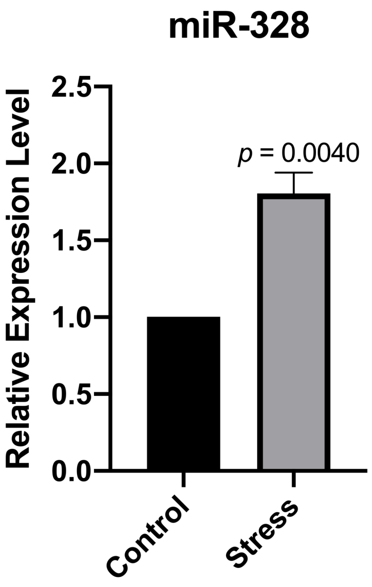

Figure S1: 24h desiccation stress significantly increased miR-328 expression in rabbit conjunctival goblet cells. n=3 for each group
