# Supplementary material for: Inhibition of microRNA-328 Increases Ocular Mucin Expression and Conjunctival Goblet Cells
Source: Biomedicines. 2023 Jan 19;11(2):287. doi: 10.3390/biomedicines11020287 (PMC9953597; doi:10.3390/biomedicines11020287)
Supplement: Supplementary file 1 [file biomedicines-11-00287-s001.zip › Supplementary tables.pdf]

Supplementary Information:

Table S1:

Primers used for plasmid construction:

	**Forward**	**Reverse**
Wild-type	CTAGCCTTTGCAAATATAGGGCCATCTTTGCAA ATATAGGGCCATCTTTGCAAATATAGGGCCATC	TCGAGATGGCCCTATATTTGCAAAGATGGCCCT ATATTTGCAAAGATGGCCCTATATTTGCAAAGG
Mutant	CTAGCCTTTGCAAATATCAAATACTCTTTGCAA ATATCAAATACTCTTTGCAAATATCAAATACTC	TCGAGAGTATTTGATATTTGCAAAGAGTATTTG ATATTTGCAAAGAGTATTTGATATTTGCAAAGG

Table S2:

Primers used in the real-time PCR experiments:

	**Forward**	**Reverse**
CREB1	CCTCCCCAGCACTTCCTACACA	TTCAGCTCCTCAATCAGCGTCT
MUC5AC	CCCCAACGTCAAGAACAACT	TCAAACAGGCAGTTCGAGTG
GAPDH	GCGACAACATCCACTTTGCC	GAGGTCGGAGTGAACGGAT
